# Dipyrone (Metamizole)-Induced Stevens-Johnson Syndrome

**DOI:** 10.7759/cureus.53122

**Published:** 2024-01-28

**Authors:** Sérgio Gomes Ferreira, Luís Fernandes, Sara Santos, Sofia Ferreira, Joana Sequeira

**Affiliations:** 1 Internal Medicine, Hospital São Sebastião, Centro Hospitalar Entre o Douro e Vouga, Santa Maria da Feira, PRT

**Keywords:** rare skin disease, drug-induced allergic response, metamizole, adverse reactions, stevens-johnson-syndrome

## Abstract

Stevens-Johnson Syndrome (SJS), a severe mucocutaneous hypersensitivity reaction primarily triggered by drugs, poses a low-incidence, high-mortality challenge. This report explores its clinical nuances and emphasizes supportive care as the mainstay of treatment. A 74-year-old female, burdened with a complex medical history, presented with a non-pruritic macular rash escalating to skin and oral mucosal involvement. A recent introduction of dipyrone (metamizole) implicated drug-induced SJS. Histopathological confirmation guided treatment involving supportive care, corticosteroids, and wound care, resulting in clinical improvement. The case underscores the significance of histopathological confirmation and thorough medication history in navigating SJS complexities, especially in patients with comorbidities like connective tissue disease. A successful multidisciplinary approach and the decision for post-discharge monitoring highlight the intricate management challenges. This case illuminates the intricate interplay of medication-induced hypersensitivity, comorbidities, and management challenges in SJS. Optimal outcomes require prompt diagnosis, trigger identification, and a multidisciplinary treatment approach, emphasizing ongoing research and clinical vigilance.

## Introduction

Stevens-Johnson syndrome (SJS) is a delayed-type hypersensitivity reaction (type IV), primarily manifesting in the mucocutaneous area. It is a severe and life-threatening condition, mainly caused by drugs and infections, and is characterized by blisters and skin detachment (with positive Nikolsky sign), with low incidence but high mortality [1,2]. There are some populations with a higher risk of developing such conditions, such as patients with connective tissue disease and older adults [3]. SJS is more common in females, with a female-to-male ratio of approximately 2:1 [4].

The skin lesions are generally non-pruritic, with two nonpalpable zones with an indistinct border ("atypical target" lesions), with only two-color zones (different from the ones seen in erythema multiforme, that usually have three rings of color). The rash usually confines itself to one area of the body, commonly the trunk, although it often affects the palms and backs of the hands, soles of the feet, and extensor surfaces [1]. Some prodromic symptoms (such as fever, malaise, and sore throat) may precede the mucocutaneous presentation [5].

The diagnostic approach includes the clinical findings and history, with the investigation of recent exposure to drugs. Definitive diagnosis of SJS is anatomopathological, confirmed through biopsy of the lesions, with some of the typical findings being keratinocyte apoptosis, epidermis separation from the dermo-epidermal junction, and a mild dermal inflammatory infiltrate of primarily T lymphocytes. Direct immunofluorescence is always negative [6]. The therapeutic approach involves promptly identifying its etiology (if drug-induced, discontinuing the causative drug). Most SJS patients receive supportive care with wound care, fluid and electrolyte management, nutritional support, temperature management, pain control, infection prevention and management, and organ support if needed [7].

## Case presentation

This report presents the case of a 74-year-old female with relevant medical history, including Sjogren's syndrome (with multiorgan involvement), Kidney Disease Improving Global Outcomes (KDIGO) stage 3b chronic kidney disease (CKD), osteomalacia secondary to corticosteroid therapy, corticosteroid-induced diabetes mellitus, dyslipidemia, and arterial hypertension. Her chronic medication included prednisolone (5 mg per day), sodium bicarbonate, potassium chloride, oral antidiabetics, nebivolol, and a statin.

**Figure 1:**
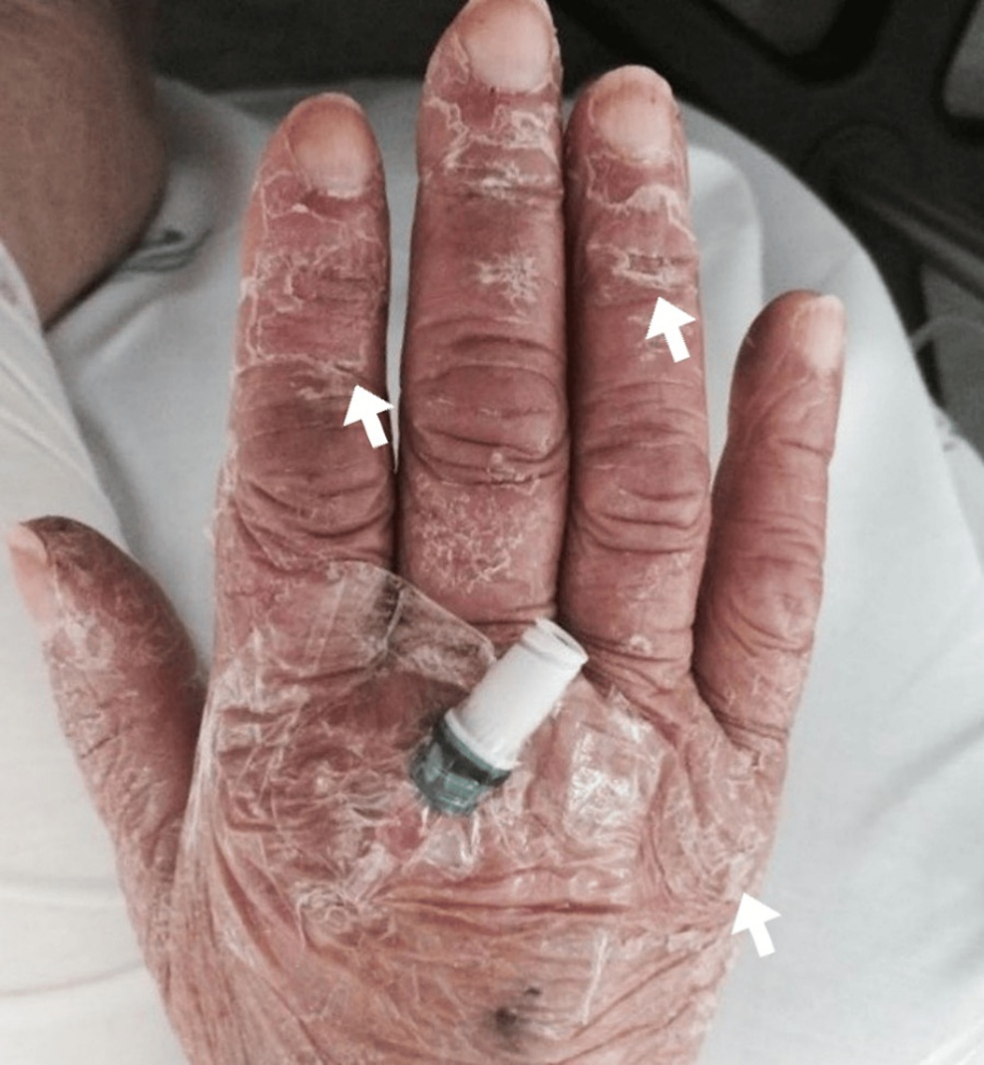
Dorsal surface of the patient's right hand showing desquamative lesions.

She presented to the emergency department with a non-pruritic macular rash evolving over two days. It started on the inner thighs and rapidly progressed to involve the skin of the dorsal surface of both hands (Figure 1) and the oral mucosa. She also complained of odynophagia and dysphagia, with no other symptoms reported. No relevant epidemiological context was identified. She mentioned the recent introduction of dipyrone (metamizole), more than a week prior to the presentation. Upon admission, she had a dispersed maculopapular rash involving the oral mucosa, with tenderness on compression, no itching, and desquamation on the inner thigh and the dorsal surface of the hands. Her vital signs were stable, there was no documented fever, and no other relevant symptoms, such as respiratory.

**Figure 2:**
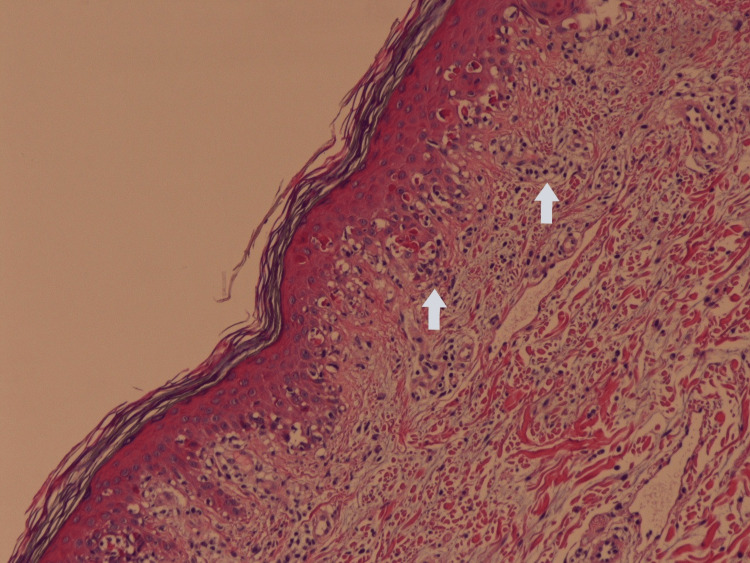
Histopathology of the patient's skin biopsy (H&E stain). There is evidence of necrosis of keratinocytes in the epidermis and lymphocyte infiltrates in the papillary dermis (arrows).

She was hospitalized with supportive treatment with continuous wound care, and daily dressing changes were promptly started. She also was on analgesic medication on demand, with no non-steroidal anti-inflammatory drugs (NSAIDs). A skin biopsy was performed before starting corticosteroids (prednisolone at 60 mg per day). Clinical improvement in pain and skin lesions (as seen later in the hospitalization) without the appearance of new lesions was evidenced. The skin biopsy revealed epidermal changes with hydropic alteration of the basal layer, no subepidermal cleft formation, moderate spongiosis, frequent apoptotic bodies, and necrosis of keratinocytes. The papillary dermis showed edema and perivascular inflammatory infiltrate consisting of lymphocytes, histiocytes, and eosinophils (Figure 2). The Dermatology Service confirmed the diagnosis of SJS, likely secondary to dipyrone (metamizole). The patient was discharged on a tapering corticosteroid regimen and scheduled for follow-up with Immunology and Allergy, and no recurrence of symptoms was evidenced.

## Discussion

Clinical presentation and diagnosis

The presented case encapsulates the classical features of SJS, a rare but severe hypersensitivity reaction primarily affecting the mucocutaneous region. The macular rash evolving into papules, vesicles, and blistering, with characteristic "target" lesions, aligns with the established clinical manifestations of SJS [1]. Notably, the involvement of the oral mucosa, the absence of pruritus, and the rapid progression of symptoms underscore the urgency and complexity of the diagnosis. The definitive confirmation of SJS through an anatomopathological examination, specifically a skin biopsy, reinforces the importance of histopathological findings in guiding accurate diagnoses. The identified epidermal changes, keratinocyte apoptosis with necrosis, and the inflammatory infiltrate observed in the papillary dermis provide a comprehensive histological profile consistent with SJS [6].

Etiology and medication-induced SJS

The patient's medical history, especially the introduction of new medications, played a pivotal role in understanding the etiology of SJS in this case. The correlation between the onset of symptoms and the recent initiation of dipyrone (metamizole), a rarely implied but documented causative drug suggests a probable drug-induced reaction [8]. The confirmation by the Dermatology Service that dipyrone (metamizole) was likely the causative agent emphasizes the importance of a thorough medication history in unraveling the trigger for SJS.

Patient comorbidities and challenges in management

The complexity of the case is further heightened by the patient's pre-existing medical conditions, including Sjogren's syndrome, CKD, and corticosteroid-induced diabetes mellitus. These comorbidities not only influence the patient's overall health but may also impact the course and management of SJS [3]. The challenge of managing a hypersensitivity reaction in the context of CKD adds another layer of complexity, requiring careful consideration of medication dosages and potential nephrotoxic effects.

Treatment and multidisciplinary approach

The therapeutic approach adopted, involving supportive treatment with careful wound care, aligns with current guidelines for managing SJS [7]. The initiation of corticosteroids reflects the severity of the case and the need for immunomodulatory intervention. Although there is little evidence, some studies support favorable outcomes in patients treated with this therapy [9]. The successful outcome, with improvement in pain and skin lesions without the emergence of new lesions during hospitalization, underscores the effectiveness of the chosen treatment strategy.

Follow-up and immunology clinic referral

The decision to discharge the patient on a tapering corticosteroid regimen and schedule follow-up in the Immunology and Allergy clinic emphasizes the importance of continued monitoring and specialized care. SJS can have long-term sequelae, and follow-up care is crucial in addressing potential complications, ensuring the resolution of symptoms, and mitigating the risk of recurrence [10].

## Conclusions

This case illustrates the intricate interplay between medication-induced hypersensitivity reactions, patient comorbidities, and the challenges in managing a rare and severe condition like SJS, especially with a not-so-common causative drug such as dipyrone (metamizole). The successful diagnosis, prompt identification of the causative agent, and the multidisciplinary treatment approach highlight the collaborative efforts required for optimal patient outcomes. The case also underscores the importance of ongoing research and clinical vigilance in understanding and managing complex presentations of SJS.

## References

[1] Hasegawa A, Abe R (2020). Recent advances in managing and understanding Stevens-Johnson syndrome and toxic epidermal necrolysis. F1000Res.

[2] Lerch M, Mainetti C, Terziroli Beretta-Piccoli B, Harr T (2018). Current perspectives on Stevens-Johnson syndrome and toxic epidermal necrolysis. Clin Rev Allergy Immunol.

[3] Ziemer M, Kardaun SH, Liss Y, Mockenhaupt M (2012). Stevens-Johnson syndrome and toxic epidermal necrolysis in patients with lupus erythematosus: a descriptive study of 17 cases from a national registry and review of the literature. Br J Dermatol.

[4] Sekula P, Dunant A, Mockenhaupt M (2013). Comprehensive survival analysis of a cohort of patients with Stevens-Johnson syndrome and toxic epidermal necrolysis. J Invest Dermatol.

[5] Duong TA, Valeyrie-Allanore L, Wolkenstein P, Chosidow O (2017). Severe cutaneous adverse reactions to drugs. Lancet.

[6] Côté B, Wechsler J, Bastuji-Garin S, Assier H, Revuz J, Roujeau JC (1995). Clinicopathologic correlation in erythema multiforme and Stevens-Johnson syndrome. Arch Dermatol.

[7] Creamer D, Walsh SA, Dziewulski P (2016). U.K. guidelines for the management of Stevens-Johnson syndrome/toxic epidermal necrolysis in adults 2016. Br J Dermatol.

[8] Sassolas B, Haddad C, Mockenhaupt M (2010). ALDEN, an algorithm for assessment of drug causality in Stevens-Johnson Syndrome and toxic epidermal necrolysis: comparison with case-control analysis. Clin Pharmacol Ther.

[9] Schneck J, Fagot JP, Sekula P, Sassolas B, Roujeau JC, Mockenhaupt M (2008). Effects of treatments on the mortality of Stevens-Johnson syndrome and toxic epidermal necrolysis: A retrospective study on patients included in the prospective EuroSCAR Study. J Am Acad Dermatol.

[10] Lee HY, Walsh SA, Creamer D (2017). Long-term complications of Stevens-Johnson syndrome/toxic epidermal necrolysis (SJS/TEN): the spectrum of chronic problems in patients who survive an episode of SJS/TEN necessitates multidisciplinary follow-up. Br J Dermatol.

